# HOW WILL AGE INCLUSIVITY SURVIVE IN AN ERA OF ANTI-DEI INITIATIVES?

**DOI:** 10.1093/geroni/igad104.0554

**Published:** 2023-12-21

**Authors:** Dana Bradley, Laura Allen

**Affiliations:** UMBC, Baltimore, Maryland, United States; University of Maryland, Baltimore County, Baltimore, Maryland, United States

## Abstract

For the first time, we have five different generations in the workplace in the United States. Generational diversity in the office is a trend that is not only expected to continue, but it is expected to grow. In that same vein, as companies continue to evolve in the way they work, it will be vital for organizations to understand, embrace and establish a diversity, equity, and inclusion (DEI) strategy in an ongoing effort to help employees of all ages feel like they are respected, valued, understood and belong in their workplace. Recent state court decisions and administrative decisions by governors suggest that DEI strategy implementations may be limited in some states. This paper explores the evolution of age inclusivity as part of DEI policies and practices. Grounded in the work of the United Nations Convention on the Rights of Older Persons, advocates have expanded their work to address ageism and ableism and ensure that society reflects persons’ lives as they age. We reviewed state-level court and administrative decisions regarding DEI initiatives across the US in 2021-23. While findings rarely note age, language in these decisions is written broadly so that outcome is unclear which subsequent court decisions. Our analysis concludes with “best practices” from the corporate sector and higher education, which emphasize systems where people on either end of the age spectrum have a more precise connection point to the decision-makers, each other, and conversations around the age at work. These strategies offer a cautious pathway forward in the US.

